# A reconstruction approach in wavelet domain for fluorescent molecular tomography via rotated sources illumination

**DOI:** 10.1186/s12938-015-0080-y

**Published:** 2015-09-30

**Authors:** Wei Zou, Jiajun Wang, Danfeng Hu, Wenxia Wang

**Affiliations:** School of Electronic and Information Engineering, Soochow University, Suzhou, 215006 China; Department of Electronic and Information Engineering, Hong Kong Polytechnic University, Kowloon, Hong Kong; School of Information Technologies, The University of Sydney, Sydney, NSW 2006 Australia

**Keywords:** Tomography, Wavelet, Reconstruction

## Abstract

**Background:**

Fluorescent molecular tomography (FMT) aims at reconstructing the spatial map of optical and fluorescence parameters from fluence measurements. Basically, solving large-scale matrix equations is computationally expensive for image reconstruction of FMT. Despite the reconstruction quality can be improved with more sources, it may result in higher computational costs for reconstruction. This article presents a novel method in the wavelet domain with rotated sources illumination.

**Methods:**

We use the finite element method for the computation of the forward model. The global inverse problem is solved based on wavelet in conjunction with principal component analysis. The iterative reconstruction is implemented with sources rotated in a certain angle. The original excitation light sources are used to reconstruct the image in the first iteration. Then, upon the sources are rotated by a certain angle, they are employed for the next iteration of reconstruction.

**Results:**

Simulation results demonstrate that our method can considerably reduce the time taken for the computation of inverse problem in FMT. Furthermore, the approach proposed is also shown to largely outperform the traditional method in terms of the precision of inverse solutions.

**Conclusions:**

Our method has the capability to locate the inclusions. The proposed method can significantly speed up the reconstruction process with the high reconstruction quality.

## Background

Over the past decade, near-infrared (NIR) biomedical optical imaging is a rapidly evolving field. It has the potential in a wide range of medical applications. The ongoing development in this area is led by the cooperation of physicians, engineers, physicists, etc. [1, 2]. Among the optical molecular imaging, fluorescent molecular tomography (FMT) is a promising tool, which is expected to have a substantial impact on the prevention and treatment of cancer and of other lethal diseases [3]. This emerging imaging modality can offer an opportunity for noninvasive visualization of biological processes at the molecular or genetic level, targeting the detection of abnormalities at the molecular stage [4, 5]. FMT depends on the perturbation of electron densities of molecules through the absorption of light at the fluorophore’s excitation wavelength. Upon radiative relaxation, fluorescent light is emitted and the fluorophore returns to its ground state with some characteristic time constant. The fluorescent photons are measured by the detectors widely spaced over the surface of the object. From these data, one can detect and map the accumulation of indocyanine green in tissue. Compared to other tomography methods, FMT offers several distinct advantages in terms of sensitivity to functional changes, safety, and cost [6]. For model-based iterative image reconstruction, the light propagation model is utilized as a predictor of measurements. Typically, the model is described by coupled partial differential equations [7]. Besides the forward model, the inversion technique is also needed for image reconstruction [8]. These techniques take into account the diffuse nature of photon propagation to achieve the spatial distribution of fluorochromes in tissues.

Considering the fact that the fluorophore is excited by the excitation light from source, the source may be an important factor for yielding the reconstructed results. Intuitionally, more sources can result in improved reconstruction results. But on the other hand, it may lead to the matrix system with larger scale and hence higher computational costs for reconstruction [9]. A model-order reduction method was proposed in [10] to reduce the computational complexity in the system matrix calculation. However, the transformation matrix needs to be constructed with the basis vectors, which possesses relatively high computational requirements. In [11], an efficient algorithm was proposed to locate and characterize the object, where the B-spline model and appropriate parameterization were utilized to reduce the number of unknowns. However, this method addresses the problem with only one object.

To accelerate inverse problem of FMT, some compression approaches have been proposed [e.g., wavelet transform, principal component analysis (PCA), etc.]. The most important feature of the wavelet transform lies in the fact that most information of the signal is contained in a small number of entries with other entries being very small and therefore can be neglected. PCA is one of the most widely used feature extraction methods, which aims to obtain the most compact representations of the high dimensional data. Some related research has been conducted in the inverse reconstruction. Ducros et al. applied compression techniques to the measurements acquired with structured illuminations [12]. This method is based on the exploitation of the wavelet transform of the measurements acquired after wavelet patterned illuminations. Correia et al. introduced a method with wavelet-based data and solution compression to improve the efficiency of image reconstruction for fluorescence diffuse optical tomography [13]. This approach preserves the resolution of the forward operator and compresses its representation. In [14], Zhang et al. proposed to use PCA to reduce the dimension of the sub weight matrix, and thus to accelerate the reconstruction process of dynamic FMT. Cao et al. solved the inverse problem based on reducing the dimension of the weight matrix with PCA [15]. Furthermore, some other fast reconstruction techniques have been investigated, including sparsity regularization based on the iterated shrinkage method [16], acceleration strategy using graphics processing unit [17, 18], and sparsity adaptive subspace pursuit method [5]. In addition, a reconstruction method using permissible region extraction strategy was proposed in [19].

Considering the compression characteristic of wavelet transform and PCA, to further speed up the reconstruction process of FMT as well as improve the precision of the inverse solutions, a new method using the wavelet-based PCA is proposed in this paper. In our method, the original excitation light sources and those rotated in a certain angle are used for iteration of image reconstruction in turn. Simulation results demonstrate that the proposed method can significantly speed up the reconstruction process and achieve high accuracy of inverse solutions.

## Methods

### Diffusion model

As it has been stated earlier, the forward model is used to predict the observable states at the measurement locations from knowledge of the excitation light source and spatial distribution of optical and fluorescent properties. The propagation of photons through a highly scattering medium with low absorption can be well described by the diffusion equation [20]. We employ the widely-used diffusion equation as a forward model that is appropriate for a variety of optical tomography schemes of tissues. Herein, the excitation field $\Phi_{x} \left( {{\mathbf{r}},\omega } \right)$ and the emission field $\Phi_{m} \left( {{\mathbf{r}},\omega } \right)$ are modelled with a pair of coupled diffusion equations as follows

1 $$ - \nabla \cdot [D_{x} \left( {\mathbf{r}} \right)\nabla \Phi_{x} \left( {{\mathbf{r}},\omega } \right)] + k_{x} \left( {{\mathbf{r}},\omega } \right)\Phi_{x} \left( {{\mathbf{r}},\omega } \right) = S_{x} \left( {{\mathbf{r}},\omega } \right) $$

2 $$ - \nabla \cdot [D_{m} \left( {\mathbf{r}} \right)\nabla \Phi_{m} \left( {{\mathbf{r}},\omega } \right)] + k_{m} \left( {{\mathbf{r}},\omega } \right)\Phi_{m} \left( {{\mathbf{r}},\omega } \right) = \alpha \left( {{\mathbf{r}},\omega } \right)\Phi_{x} \left( {{\mathbf{r}},\omega } \right) $$

where the first equation depicts the transport of the excitation photons and the second one describes the excitation and transport of the fluorescent photons; $\nabla$ is the grad operator, $S_{x} \left( {{\mathbf{r}},\omega } \right)$ is the source term for the excitation light; $D_{x,m} \left( {\mathbf{r}} \right)$ and $k_{x,m} \left( {{\mathbf{r}},\omega } \right)$ denote the diffusion and decay coefficients at the excitation and emission wavelengths, respectively; $\alpha$ is the emission source coefficient. They are defined by:

3 $$ D_{x,m} \left( {\mathbf{r}} \right) = \frac{1}{{3[\mu_{ax,mi} \left( {\mathbf{r}} \right) + \mu_{ax,mf} \left( {\mathbf{r}} \right) + \mu^{\prime}_{sx,m} \left( {\mathbf{r}} \right)]}} $$

4 $$ k_{x,m} \left( {{\mathbf{r}},\omega } \right) = \frac{i\omega }{c} + \mu_{ax,mi} \left( {\mathbf{r}} \right) + \mu_{ax,mf} \left( {\mathbf{r}} \right) $$

5 $$ \alpha \left( {{\mathbf{r}},\omega } \right) = \frac{{\eta \mu_{axf} \left( {\mathbf{r}} \right)}}{{1 - i\omega \tau \left( {\mathbf{r}} \right)}} $$

where $\mu_{ax,mi} \left( {\mathbf{r}} \right)$ represent the absorption coefficients due to non-fluorescing chromophore; $\mu_{ax,mf} \left( {\mathbf{r}} \right)$ represent the absorption coefficients due to fluorophore; $\mu^{\prime}_{sx,m} \left( {\mathbf{r}} \right)$ denote the isotropic scattering coefficients; fluorescence parameters $\eta$ and $\tau \left( {\mathbf{r}} \right)$ denote the fluorescence quantum efficiency and fluorescence lifetime, respectively; $c$ is the speed of light in the media; $i$ is the imaginary unit; $\omega$ stands for the angular modulation frequency of the input signal.

Here, we make use of the popular Robin boundary conditions for a bounded domain $\Omega$, which take the form as

6 $$ \Phi_{x} \left( {{\mathbf{r}},\omega } \right) + 2A_{x} \left( {\mathbf{r}} \right)D_{x} \left( {\mathbf{r}} \right){\mathbf{n}}\left( {\mathbf{r}} \right) \cdot \nabla \Phi_{x} \left( {{\mathbf{r}},\omega } \right) = 0 $$

7 $$ \Phi_{m} \left( {{\mathbf{r}},\omega } \right) + 2A_{m} \left( {\mathbf{r}} \right)D_{m} \left( {\mathbf{r}} \right){\mathbf{n}}\left( {\mathbf{r}} \right) \cdot \nabla \Phi_{m} \left( {{\mathbf{r}},\omega } \right) = 0 $$

where ${\mathbf{n}}$ is the outer normal to the boundary, and $A_{x,m} \left( {\mathbf{r}} \right)$ is a parameter modelling internal reflection at the boundary.

### Finite element approximation of the forward model

Like most others working in FMT, we are currently using the finite element method (FEM) for the computation of the forward model. FEM is versatile especially in regard to complex geometries and for modelling boundary effects [21]. In principle, FEM can be applied to any partial differential equations model of the transport process. In the FEM framework, the computational domain is discretized to a mesh with *P* elements and *N* vertex nodes [22]. The solution $\Phi_{x,m}$ is approximated by the piecewise function $\Phi_{x,m} = \sum\nolimits_{i}^{N} {{\varvec{\Phi}}_{xi,mi} \varphi_{i} }$, with locally supported basis functions $\varphi_{i}$ ($i = 1,2, \ldots ,N$).

Suppose $V_{0}^{h} = span\left\{ {\varphi_{j} } \right\}_{j = 1}^{N}$ and thus $v_{h} = \sum\nolimits_{k = 1}^{N} {c_{k} \varphi_{k} }$. Let $u_{h} = \sum\nolimits_{j = 1}^{N} {\Phi_{j} \varphi_{j} }$. To yield the weak solutions of the forward equations, we rewrite Eqs. (1) and (2) by the formulation as

8 $$ a_{{\Omega_{h} }} (u_{h} ,v_{h} )_{x,m} = (f_{x,m} ,v_{h} )_{{\Omega_{h} }} $$

where

9 $$ a_{{\Omega_{h} }} (u_{h} ,v_{h} )_{x,m} = \iint\limits_{{\Omega_{h} }} {[D_{x,m} (\nabla u_{h} \cdot \nabla v_{h} ) + k_{k,m} u_{h} v_{h} ]d\Omega } + \int\limits_{{\Gamma_{h} }} {b_{x,m} u_{h} v_{h} ds} $$

10 $$ (f_{x,m} ,v_{h} )_{{\Omega_{h} }} = \iint\limits_{{\Omega_{h} }} {f_{x,m} v_{h} d\Omega } $$

11 $$ f_{x} = S_{x} ,\quad f_{m} = \beta \Phi_{x} $$

with the bounded domain $\Omega_{h}$ and its boundary $\Gamma_{h}$.

Equation (8) can be written by the matrix formulation

12 $$ {\mathbf{A}}_{x,m} {\varvec{\Phi}}_{x,m} = {\mathbf{S}}_{x,m} $$

where

13 $$ {\mathbf{S}}_{x,m} = \left[ {\begin{array}{*{20}c} {(f_{x,m} ,\varphi_{1} )_{{\Omega_{h} }} } \\ \vdots \\ {(f_{x,m} ,\varphi_{N} )_{{\Omega_{h} }} } \\ \end{array} } \right] $$

14 $$ {\mathbf{A}}_{x,m} = \left[ {\begin{array}{*{20}c} {a_{{\Omega_{h} }} (\varphi_{1} ,\varphi_{1} )_{x,m} } & \cdots & {a_{{\Omega_{h} }} (\varphi_{N} ,\varphi_{1} )_{x,m} } \\ \vdots & {} & \vdots \\ {a_{{\Omega_{h} }} (\varphi_{1} ,\varphi_{N} )_{x,m} } & \cdots & {a_{{\Omega_{h} }} (\varphi_{N} ,\varphi_{N} )_{x,m} } \\ \end{array} } \right]. $$

The matrices ${\mathbf{A}}_{x,m}$ have elements

15 $$ a_{{\Omega_{h} }} (\varphi_{i} ,\varphi_{j} )_{x,m} = \iint\limits_{{\Omega_{h} }} {D_{x,m} \nabla \varphi_{i} \cdot \nabla \varphi_{j} d\Omega + \iint\limits_{{\Omega_{h} }} {k_{x,m} \varphi_{i} \varphi_{j} d\Omega }} + \int\limits_{{\Gamma_{h} }} {b_{x,m} \varphi_{i} \varphi_{j} ds} . $$

Combining Eqs. (12) and (15), the forward equations within the FEM scheme become

16 $$ \left( {{\mathbf{D}}_{x} + {\mathbf{K}}_{x} + {\mathbf{B}}_{x} } \right){\varvec{\Phi}}_{x} = {\mathbf{S}}_{x} $$

17 $$ \left( {{\mathbf{D}}_{m} + {\mathbf{K}}_{m} + {\mathbf{B}}_{m} } \right){\varvec{\Phi}}_{m} = {\mathbf{S}}_{m} $$

where

18 $$ D_{ij} = \iint\limits_{{\Omega_{h} }} {D_{x,m} \nabla \varphi_{i} \cdot \nabla \varphi_{j} d\Omega } $$

19 $$ K_{ij} = \iint\limits_{{\Omega_{h} }} {k_{x,m} \varphi_{i} \varphi_{j} d\Omega } $$

20 $$ B_{ij} = \int\limits_{{\Gamma_{h} }} {b_{x,m} \varphi_{i} \varphi_{j} ds} . $$

### Inverse problem

The inverse problem of FMT consists in estimating the optical parameters and fluorescent properties of the tissue by using the measured data as described earlier [23]. To generally pose the inverse problem, we first define the forward mapping as $F$. Therefore, the inverse problem reads

21 $$ x = F^{ - 1} \left( y \right) $$

where $y$ denotes boundary measurement, and $x$ denotes optical or fluorescent properties.

The above non-linear problem can be linearized. To proceed, we can expand about $x_{0}$ in a Taylor series. Neglecting the higher order terms, we thus arrive at the linear problem as

22 $$ y - y_{0} = {\mathbf{J}}\left( {x - x_{0} } \right) $$

where ${\mathbf{J}}$ is the Jacobian of the forward mapping.

Due to the fact that the inverse reconstruction problem is ill-posed and underdetermined, we introduce the Moore–Penrose inversion in conjunction with Tikhonov regularization, leading to the following formula:

23 $$ x - x_{0} = \left( {{\mathbf{J}}^{T} {\mathbf{J}} +\upxi{\mathbf{I}}} \right)^{ - 1} {\mathbf{J}}^{T} \left( {y - y_{0} } \right) $$

where ${\mathbf{I}}$ represents the identity matrix, and $\upxi$ acts as a regularization parameter.

Equation (23) can be written in a succinct matrix form by

24 $$ {\mathbf{K}}\Delta {\mathbf{x}} = {\mathbf{b}} $$

where we define ${\mathbf{K}} = \left( {{\mathbf{J}}^{T} {\mathbf{J}} + \xi {\mathbf{I}}} \right)$ and ${\mathbf{b}} = {\mathbf{J}}^{T} \Delta {\mathbf{y}}$.

### Image reconstruction with the wavelet-based principal component analysis

We solve the inverse problem in the wavelet domain. To this aim, we take the wavelet transform on both sides of Eq. (24)

25 $$ {\hat{\mathbf{K}}}\Delta {\hat{\mathbf{x}}} = {\hat{\mathbf{b}}} $$

where ${\hat{\mathbf{K}}} = {\mathbf{W}}_{{\mathbf{b}}} {\mathbf{KW}}_{{\mathbf{x}}}^{T}$, $\Delta {\hat{\mathbf{x}}} = {\mathbf{W}}_{{\mathbf{x}}} \Delta {\mathbf{x}}$, ${\hat{\mathbf{b}}} = {\mathbf{W}}_{{\mathbf{b}}} {\mathbf{b}}$. However, the level-by-level implementation scheme in the conventional wavelet-based reconstruction method [24] not only is computationally expensive but also causes information lost in the system matrix of the reconstruction problem [25], which inevitably deteriorates the final reconstruction quality. In order to circumvent that problem, we propose to solve the global inverse problem as Eq. (24) based on wavelet in conjunction with the PCA instead of the level-by-level wavelet transform scheme. To this aim, we briefly present the PCA principles. It is well known that PCA performs a dimensionality reduction by searching for a projection matrix with a small number of eigenvectors with respect to the largest eigenvalues. Assume that ${\mathbf{L}}$ is the covariance matrix of the given matrix ${\mathbf{K}}$, that is,

26 $$ {\mathbf{L}} = E\left\{ {\left[ {{\mathbf{K}} - E\left( {\mathbf{K}} \right)} \right]\left[ {{\mathbf{K}} - E\left( {\mathbf{K}} \right)} \right]^{T} } \right\} $$

${\mathbf{L}}$ can be diagonalized via

27 $$ {\mathbf{L}} = {\varvec{\Psi}}\Lambda {\varvec{\Psi}}^{T} $$

where $\Lambda$ is a diagonal matrix consisting of the eigenvalues of ${\mathbf{L}}$, and ${\varvec{\Psi}}$ is the matrix of eigenvectors of ${\mathbf{L}}$.

Thus the principal components of the matrix ${\mathbf{K}}$ can be achieved by

28 $$ {\tilde{\mathbf{K}}} = {\mathbf{\Psi K}}. $$

Multiplying (24) from the left with ${\varvec{\Psi}}$, one has

29 $$ {\tilde{\mathbf{K}}}\Delta {\mathbf{x}} = {\tilde{\mathbf{b}}} $$

where $$ {\tilde{\mathbf{b}}} = {\mathbf{\Psi b}} $$.

Keeping the first $q$ largest principal components, we can obtain a new matrix equation with reduced scale, namely

30 $$ {\tilde{\mathbf{K}}}_{q} \Delta {\mathbf{x}} = {\tilde{\mathbf{b}}}_{q} . $$

Therefore, the global matrix system as Eq. (24) can be approximately solved with the reduced-scale matrix system according to PCA.

The inverse reconstruction with the wavelet-based PCA is summarized in “Algorithm 1”.

### Algorithm 1

1. Take wavelet transform with respect to **K** and **b** in Eq. (24) to achieve the approximation components ${\hat{\mathbf{K}}}_{1}$ and ${\hat{\mathbf{b}}}_{1}$;
2. Solve ${\hat{\mathbf{K}}}_{1} \Delta {\hat{\mathbf{x}}}_{1} = {\hat{\mathbf{b}}}_{1}$ with PCA;
3. Prolongate $\Delta {\hat{\mathbf{x}}}_{1}$ by padding zeros to achieve an initial guess for $\Delta {\hat{\mathbf{x}}}$ at the original resolution, i.e., $\Delta {\hat{\mathbf{x}}}^{\left( 0 \right)} = \left[ {\Delta {\hat{\mathbf{x}}}_{1}^{T} ,{\mathbf{0}}^{T} } \right]^{T}$;
4. Solve ${\mathbf{K}}\Delta {\mathbf{x}} = {\mathbf{b}}$ with the initial guess $\Delta {\mathbf{x}}^{\left( 0 \right)} = {\mathbf{W}}_{{\mathbf{x}}}^{ \, T} \Delta {\hat{\mathbf{x}}}^{\left( 0 \right)}$.

### Iteration based on the strategy of excitation light sources rotation

**Fig. 1:**
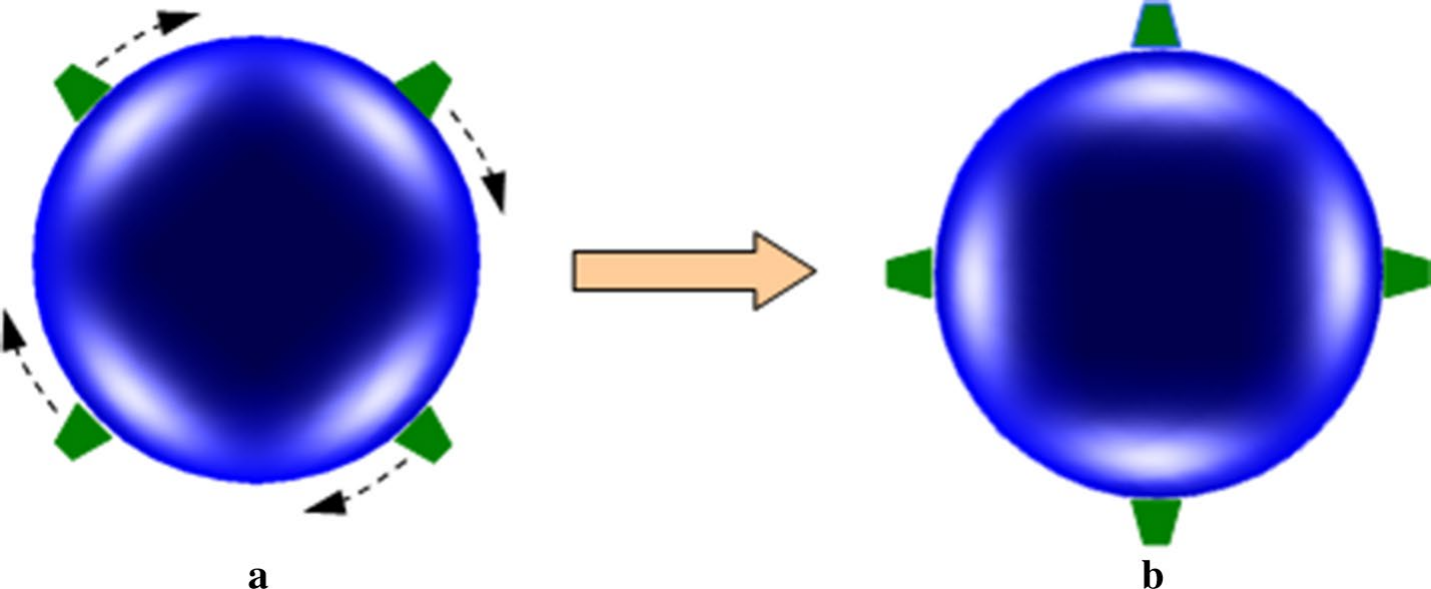
Illustrative explanation of the strategy of sources rotation. **a** Sources before rotation, and **b** sources after rotation

The tomographic imaging involves placing sources and detectors over the available surface of the tissue. Basically, the excitation light sources are arranged at the fixed positions during the process of image reconstruction. By means of increased number of sources, the image quality can be improved. However, such a strategy may result in the matrix system with larger scale and hence higher computational complexity. Although we can reduce the number of sources to safe the computation time, the information for image reconstruction will decrease, which may lead to the poor quality of reconstruction. As a result, there exists a contradiction between the reconstruction accuracy and the computational requirements. In order to address this tradeoff, we propose a new strategy for iterative calculation. In such a strategy, the original excitation light sources are used to reconstruct the image in the first iteration. Then, upon the sources are rotated by a certain angle, they are employed for the second iteration of reconstruction. This means that the whole iterative reconstruction is performed using the sources with different rotation angles in turn. This process is repeated until some stopping criteria are satisfied. This strategy is motivated by the fact that the excitation light sources from different angles can provide more information than those from some fixed angle during the iteration process, and thus the quality of reconstructed results can be improved. In our method, the number of excitation light sources is not increased, and thus it will not lead to higher computational cost. Moreover, the iterative results from the sources with one angle can provide a good initial guess for the next iteration from the sources with other angle. In this way, the precision of solutions can be improved with rotation of the lights. However, if the rotation angle of sources is too small, it may provide quite limit information for reconstruction. On the contrary, large rotation angle may lead to the superposition between the original and rotated sources. In such a case, it is unable to provide additional information for iterative reconstruction. Suppose the sources are distributed around the circumference of the tissue with equal angle between each source. To overcome those difficulties, in our work, the rotation angle is set as a half of the angle between each source. This strategy can be schematically illustrated as in Fig. 1.

For derivation of the algorithm, we minimize the residual error between the predicted data and measured data to acquire the solution to the reconstruction problem by

31 $$ M\left( {\mathbf{x}} \right) = \left\| {{\mathbf{y}} - F\left( {\mathbf{x}} \right)} \right\| $$

where $M\left( {\mathbf{x}} \right)$ is the objective function, ${\mathbf{y}}$ is the measured data, and $F\left( {\mathbf{x}} \right)$ is the predicted data with regard to a forward model. Let us suppose that $\beta$ is a half of the angle between each source, and thus the resulting reconstruction algorithm is summarized in “Algorithm 2”.

### Algorithm 2

1. **Initialize**$$ {\mathbf{x}} = {\mathbf{x}}_{0} $$, *i* = 0;
2. Repeat
3. $$ \theta = i \cdot \beta $$;
4. Compute $\Delta {\mathbf{y}}$ and ${\mathbf{J}}$ at **x** based on the excitation light sources with the rotation angle $\theta$;
5. *i* = *i* + 1;
6. Solve Eq. (24) with “Algorithm 1”;
7. Update **x** with $$ {\mathbf{x}} = {\mathbf{x}} + \Delta {\mathbf{x}} $$;
8. Compute the objective function $M\left( {\mathbf{x}} \right)$ with the current **x** by Eq. (31);
9. **Until**$$ M\left( {\mathbf{x}} \right) < \delta $$
10. **Outputx**.

### Simulation results and discussion

In this section we performed simulation study using different phantoms to test the performance of our algorithm and the obtained results. The forward model as Eqs. (1) and (2) is used to simulate the measured data. In order to better simulate the realistic conditions, we add Gaussian noise with a signal-to-noise ratio of 10 dB to the calculated data. Actually, large regularization parameter may lead to low contrast and resolution of the image, while small parameter can result in increased contrast and resolution. However, small parameter also increases the high frequency noise in the image [26]. The regularization parameter $\xi$ is set to 0.001 in the simulations for better results after a lot of simulations. The termination criterion $\delta$ is set to 0.02.

**Fig. 2:**
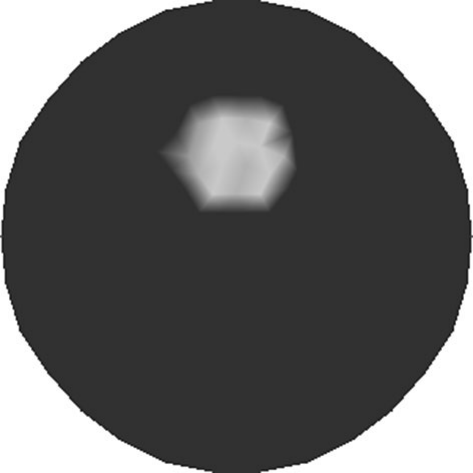
Simulated phantom with one inclusion. The value of absorption coefficient $\mu_{axf}$ of the inclusion is 0.4 mm^−1^, and the value of absorption coefficient $\mu_{axf}$ of the background is 0.06 mm^−1^

In the first example, verification of the performance of the proposed method is investigated using the test phantom containing one inclusion as indicated in Fig. 2. Four excitation light sources are uniformly distributed around the simulated phantom. The measurements are sampled by thirty detectors uniformly placed on the boundary of the phantom.

**Fig. 3:**
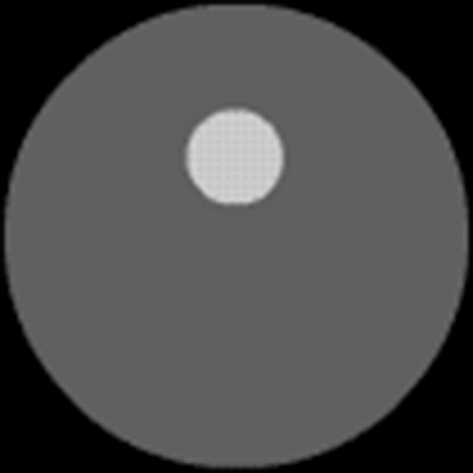
Prior image for phantom with one inclusion. The prior image is utilized to guide the generation of the adaptively refined mesh for one-inclusion reconstruction

**Fig. 4:**
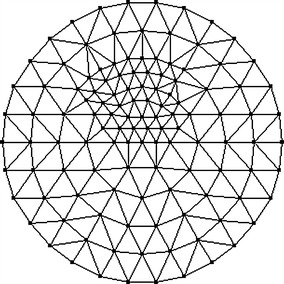
Adaptively refined mesh for reconstruction of one-inclusion phantom. The adaptively refined mesh contains 122 nodes and 212 triangular elements

To reduce the computational requirements without significant reduction of image resolution, we compute the reconstructions based on the mesh that is adaptively refined with respect to the a priori image as portrayed in Fig. 3. Figure 4 displays the mesh containing 122 nodes and 212 triangular elements.

The details for optical and fluorescent parameters in different areas of the test phantom are provided in Table 1. In order to compare the reconstructed object with the true one, we define an image quality metric by introducing the mean square error (MSE), given as

32 $$ \text{MSE} = \frac{1}{N}\sum\limits_{i = 1}^{N} {[x^{rec} \left( i \right) - x^{act} \left( i \right)]^{2} } $$

where the superscript *rec* denotes the values obtained using reconstruction algorithms, and *act* denotes the actual distribution of the optical or fluorescent parameters which is used to generate the synthetic image data set.

**Table 1:** Optical parameters used for one-inclusion phantom

$\mu_{axf} \left( {{\text{mm}}^{ - 1} } \right)$	$\mu_{axi} \left( {{\text{mm}}^{ - 1} } \right)$	$\mu^{\prime}_{sx} ({\text{mm}}^{ - 1} )$	$\mu_{amf} \left( {{\text{mm}}^{ - 1} } \right)$	$\mu_{ami} \left( {{\text{mm}}^{ - 1} } \right)$	$\mu^{\prime}_{sm} ({\text{mm}}^{ - 1} )$	$\phi$	$\tau \left( {\text{ns}} \right)$
Inclusion							
0.4	0.03	4.0	0.2	0.02	3.0	0.2	0.6
Background							
0.06	0.03	4.0	0.005	0.02	3.0	0.2	0.6

**Fig. 5:**
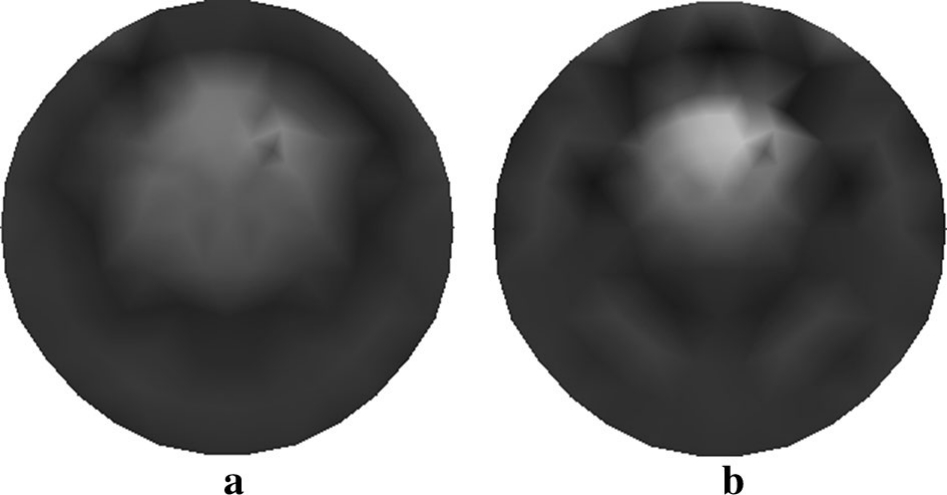
Reconstructed images of absorption coefficient $\mu_{axf}$ for one-inclusion phantom. **a** Reconstructed image with two sources, and **b** reconstructed image with four sources

The reconstructed images of $\mu_{axf}$ for one-inclusion phantom with two sources and four sources are depicted in Fig. 5a, b, respectively. Both of them are obtained without using the wavelet-based PCA. The results presented in Fig. 5 can be explained by considering that reconstruction with the increasing number of sources can enhance the quality of image, whereas the time requirements for reconstruction may increase.

**Fig. 6:**
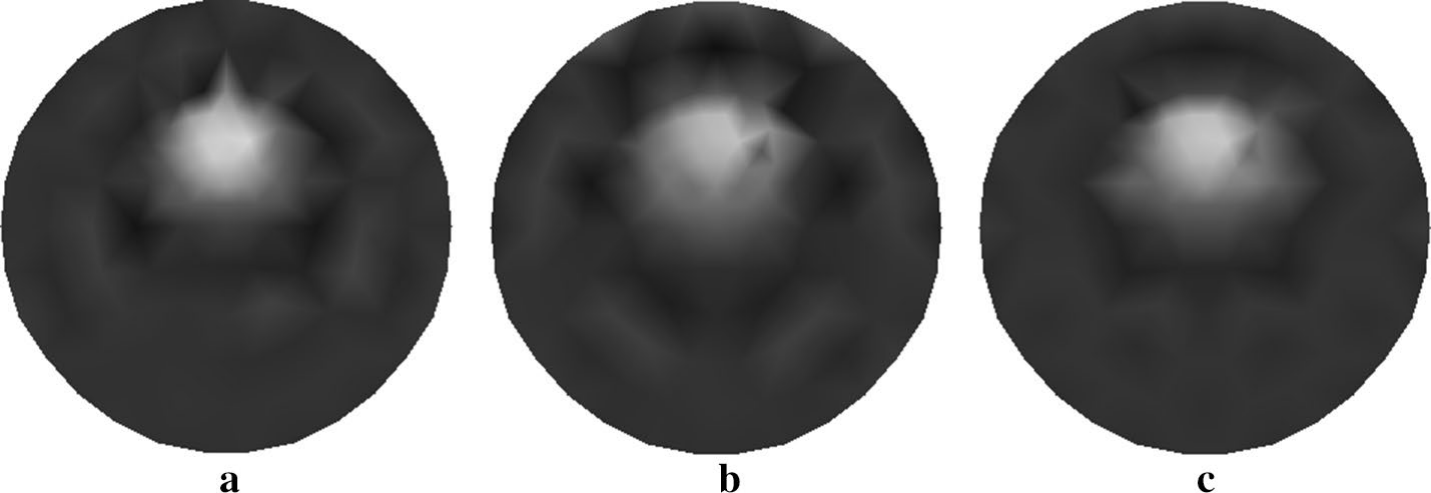
Reconstructed images of absorption coefficient $\mu_{axf}$ for phantom with one inclusion. **a** Reconstructed image based on the proposed method with four sources, **b** reconstructed image based on the traditional method with four sources, and **c** reconstructed image based on the traditional method with eight sources

In Fig. 6 we show the resulting reconstructions using the different algorithms. Figure 6a displays the reconstructed result using the proposed method with four sources. Figure 6b, c depict the traditional reconstructed result with four sources and that with eight sources, respectively. We see that the method proposed is capable of yielding the reconstructed target with improved contrast and contour comparatively to the traditional method.

We demonstrate the benefits of the proposed method by comparing the performance of our method to the traditional method. For quantitative validation, the performance of reconstructions in terms of the computation time and MSE is tabulated in Table 2. We remark, that the computation time for the proposed algorithm is much faster than the traditional method, which demonstrates that our method is time efficient. Although the increased number of sources can improve the quality of reconstruction, it will slow down the speed of reconstruction. In addition, the MSE of the proposed method is smaller than that of the other compared method. Therefore, the above results suggest that the algorithm proposed can substantially speed up the reconstruction process and possess high accuracy.

**Table 2:** Method performance comparison for phantom with one inclusion

Method	Our algorithm	Conventional method with four sources	Conventional method with eight sources
Computation time	150 (s)	215 (s)	401 (s)
MSE	4.267 × 10^−4^	4.794 × 10^−4^	4.663 × 10^−4^

**Fig. 7:**
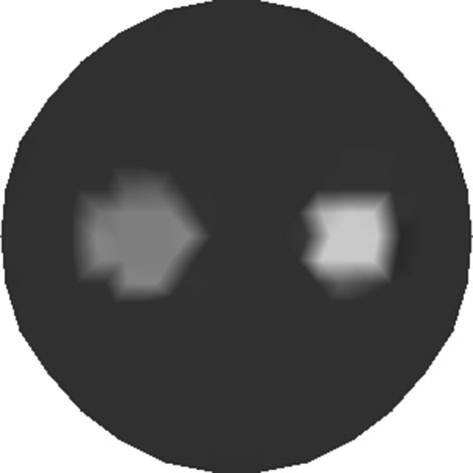
Simulated phantom with two inclusions. The value of low absorption coefficient $\mu_{axf}$ of the inclusion is 0.3 mm^−1^, the value of high absorption coefficient $\mu_{axf}$ of the inclusion is 0.4 mm^−1^, and the value of absorption coefficient $\mu_{axf}$ of the background is 0.06 mm^−1^

The phantom for the second test case is shown in Fig. 7. It consists of two inclusions of different shapes. As before, the phantom is illuminated by four equally spaced sources located on its boundary. The detector readings are obtained from 30 different points from the boundary of the circular domain. The distance between the successive detector positions is the same through the boundary.

**Fig. 8:**
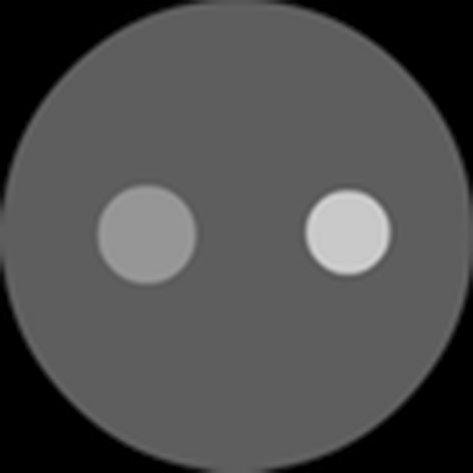
Prior image for phantom with two inclusions. The prior image is utilized to guide the generation of the adaptively refined mesh for two-inclusion reconstruction

**Fig. 9:**
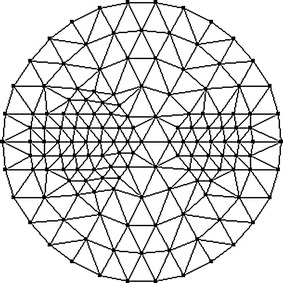
Adaptively refined mesh for reconstruction of two-inclusion phantom. The adaptively refined mesh contains 148 nodes and 264 triangular elements

Figure 8 displays the a priori image as a guidance for generation of the adaptively refined mesh. The resulting mesh with 148 nodes and 264 triangular elements is depicted in Fig. 9. Table 3 lists the values of optical and fluorescent parameters of the simulated phantom.

**Table 3:** Optical parameters used for two-inclusion phantom

$\mu_{axf} \left( {{\text{mm}}^{ - 1} } \right)$	$\mu_{axi} \left( {{\text{mm}}^{ - 1} } \right)$	$\mu^{\prime}_{sx} ({\text{mm}}^{ - 1} )$	$\mu_{amf} \left( {{\text{mm}}^{ - 1} } \right)$	$\mu_{ami} \left( {{\text{mm}}^{ - 1} } \right)$	$\mu^{\prime}_{sm} ({\text{mm}}^{ - 1} )$	$\phi$	$\tau \left( {\text{ns}} \right)$
Inclusions							
0.3, 0.4	0.03	4.0	0.02, 0.03	0.02	3.0	0.2	0.6
Background							
0.06	0.03	4.0	0.003	0.02	3.0	0.2	0.6

**Fig. 10:**
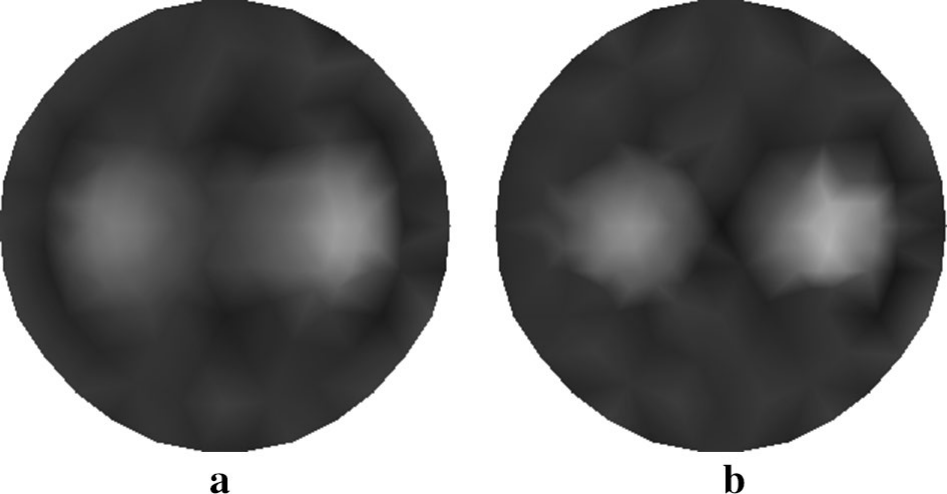
Reconstructed images of absorption coefficient $\mu_{axf}$ for two-inclusion phantom. **a** Reconstructed image with two sources, and **b** reconstructed image with four sources

In Fig. 10 we show the reconstructed images of $\mu_{axf}$ for two-inclusion phantom with 2 sources (see Fig. 10a) and that with four sources (see Fig. 10b). We also notice that one can obtain better reconstructed results with increasing sources. Nevertheless, reconstruction with more sources may lead to a heavy computation burden.

**Fig. 11:**
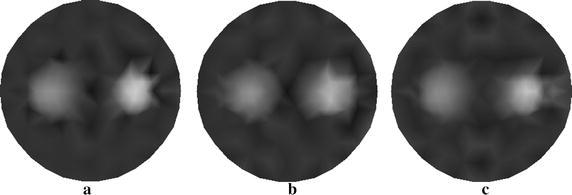
Reconstructed images of absorption coefficient $\mu_{axf}$ for phantom with two inclusions. **a** Reconstructed image based on the proposed method with four sources, **b** reconstructed image based on the traditional method with four sources, and **c** reconstructed image based on the traditional method with eight sources

The reconstruction from our method with four sources is shown in Fig. 11a and those from the traditional method are depicted in Fig. 11b, c. Particularly, the reconstructed images are obtained with four sources (see Fig. 11b) and eight sources (see Fig. 11c).

We find that the contrast of image can be enhanced with our method. The reconstruction with more sources can improve the reconstruction accuracy while result in a heavy computation burden. More importantly, we also note that the proposed method can improve the quality of reconstruction with more accurate shape and position of both targets.

We provide the quantitative comparisons of different reconstructions presented in Table 4. As can be clearly seen, improvement in quality of reconstruction can be achieved by proposed algorithm. Additionally, it is evident from Table 4 that our method requires less reconstruction time as compared with the traditional method. Therefore, the main conclusion we can draw from these simulation studies is that the approach proposed has comparable computational efficiency to the traditional method and high capability to achieve accurate reconstruction.

**Table 4:** Method performance comparison for phantom with two inclusions

Method	Our algorithm	Conventional method with four sources	Conventional method with eight sources
Computation time	209 (s)	283 (s)	537 (s)
MSE	2.406 × 10^−4^	2.817 × 10^−4^	2.714 × 10^−4^

**Fig. 12:**
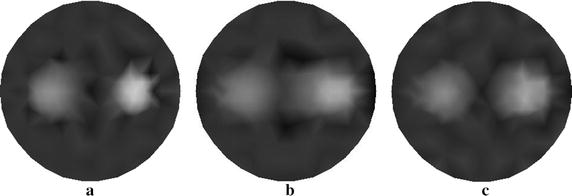
Reconstructed images of absorption coefficient $\mu_{axf}$ using different methods. **a** Reconstructed image based on the proposed method, **b** reconstructed image based on the wavelet method, and **c** reconstructed image based on the PCA method

To illustrate the superiorities of the proposed algorithm, we show the reconstructed results from the different algorithms (see Fig. 12). Figure 12a–c display the reconstructed images with the proposed approach, wavelet method, and PCA method, respectively. Table 5 summarizes the quantitative performance of reconstruction. From Table 5, it can be clearly seen that the proposed algorithm has better performance on accuracy and speed of reconstruction than algorithms only using wavelet method or PCA method.

**Table 5:** Performance comparison of different methods

Method	Our algorithm	Wavelet method	PCA method
Computation time	209 (s)	231 (s)	217 (s)
MSE	2.406 × 10^−4^	2.639 × 10^−4^	2.823 × 10^−4^

**Fig. 13:**
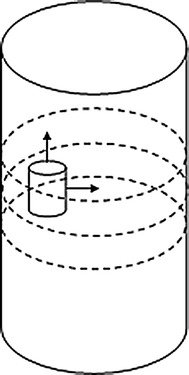
Simulated phantom for 3D reconstruction. The phantom of radius 10 mm and height 40 mm with a uniform background of $\mu_{axf} = 0.005$ mm^−1^ is located at $x = 10$ mm, $y = 0$ mm and $z = 20$ mm. The small cylindrical inclusion has a radius of 2 mm and height 6 mm with $\mu_{axf} = 0.01$ mm^−1^. The inclusion is located at $x = 5$ mm, $y = 0$ mm, and $z = 20$ mm. The *dashed curves* represent the measurement planes, at $z = 15$ mm, $z = 20$ mm, $z = 25$ mm

**Fig. 14:**
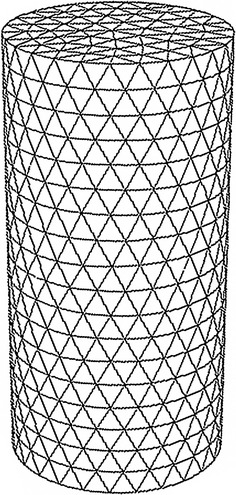
Mesh for 3D image reconstruction. Mesh for 3D image reconstruction contains 858 nodes and 3208 tetrahedral elements

**Fig. 15:**
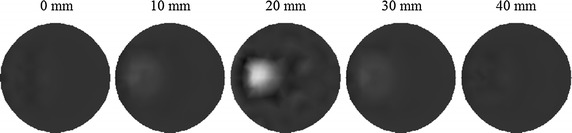
Reconstructed images based on the proposed algorithm. The *right-hand side* corresponds to the *top* of the cylinder ($z = 40$ mm), and the *left-hand side* corresponds to the *bottom* of the cylinder ($z = 0$ mm), with each slice representing a 10 mm increment

**Fig. 16:**
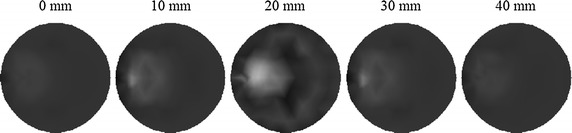
Reconstructed images based on the traditional method. The *right-hand side* corresponds to the *top* of the cylinder ($z = 40$ mm), and the *left-hand side* corresponds to the *bottom* of the cylinder ($z = 0$ mm), with each slice representing a 10 mm increment

To validate the proposed approach in the 3D case, the methods previously defined for triangular elements are extended to tetrahedral elements. The integration of products of shape functions over the volume of the elements, and surface integrals over a side of the element is performed by numerical integration rules. Herein, a cylindrical phantom as illustrated in Fig. 13 is utilized for 3D simulations. A small cylindrical inclusion is suspended in this phantom. The dashed curves represent the planes of measurement. Six sources and sixteen measurements are employed for each plane. The data are collected in all three measurement planes. The mesh for 3D reconstruction containing 3208 tetrahedral elements as well as 858 nodes is shown in Fig. 14. Figures 15 and 16 display the 3D reconstructed images based on the proposed approach and the traditional method, respectively. These are 2D cross sections through the reconstructed 3D images. The quantitative performance of the above two methods is given in Table 6 to further evaluate the reconstruction quality. As one can see from Table 6, our proposed algorithm can also significantly speed up the reconstruction process and improve the quality of reconstruction in the 3D case.

**Table 6:** Performance comparison of 3D reconstruction methods

Method	Computation time	MSE
Our algorithm	2503 (s)	3.361 × 10^−3^
Conventional method	3647 (s)	3.859 × 10^−3^

**Fig. 17:**
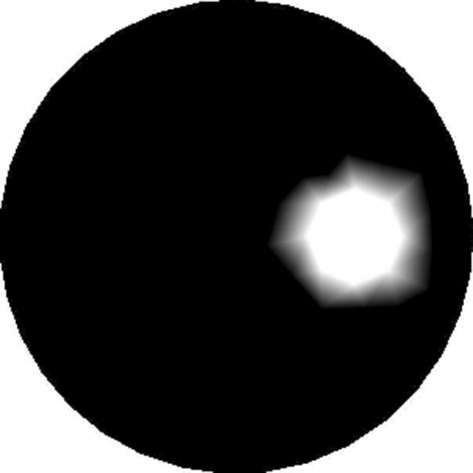
Simulated phantom based on Monte Carlo method. The value of absorption coefficient $\mu_{axf}$ of the inclusion is 0.06 mm^−1^, and the value of absorption coefficient $\mu_{axf}$ of the background is 0.025 mm^−1^

**Fig. 18:**
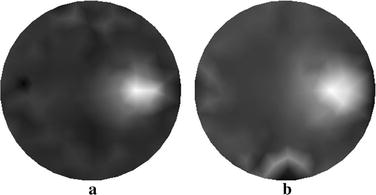
Reconstructed results with different methods. **a** Reconstructed image based on the proposed method, and **b** reconstructed image based on the conventional method

Finally, we test the reconstruction algorithms with the Monte Carlo method. As most commonly used stochastic technique, Monte Carlo method is regarded as gold standard for modelling the light propagation and has a long pedigree in transport theory. We utilize the Monte Carlo method to generate the measurement data, which is employed to reconstruct the image of FMT. Figure 17 shows the model for reconstruction and Fig. 18 shows the corresponding reconstructed results with four sources. The reconstructed results are obtained from the proposed algorithm (see Fig. 18a) and the conventional method (see Fig. 18b). The quantitative performance is listed in Table 7, from which we can also see that both the speed and precision of the reconstruction can be improved with the proposed algorithm.

**Table 7:** Method performance comparison based on Monte Carlo simulation

Method	Our algorithm	Conventional method
Computation time	274 (s)	354 (s)
MSE	7.602 × 10^−4^	8.976 × 10^−4^

## Conclusions

In this work, we have developed a highly efficient method for image reconstruction of FMT by means of wavelet-based PCA combining the new strategy for iterative calculation. During the process of reconstruction, the excitation light sources are rotated for each iteration. The proposed algorithm is tested by numerical experiments based on simulated data obtained both from the deterministic forward model and the stochastic Monte Carlo simulation. We see from the results shown in the previous sections that our method can considerably reduce the time taken for the computation of inverse problem in FMT. Furthermore, the approach proposed is also shown to largely outperform the traditional method in terms of the precision of inverse solutions. Therefore, we expect that, this study might be used both to improve current reconstruction methods and also as a guidance for clinical studies.

## Abbreviations

NIR: near-infrared
FMT: fluorescent molecular tomography
PCA: principal component analysis
FEM: finite element method
MSE: mean square error
